# A *Drosophila* Model of High Sugar Diet-Induced Cardiomyopathy

**DOI:** 10.1371/journal.pgen.1003175

**Published:** 2013-01-10

**Authors:** Jianbo Na, Laura Palanker Musselman, Jay Pendse, Thomas J. Baranski, Rolf Bodmer, Karen Ocorr, Ross Cagan

**Affiliations:** 1 Department of Developmental and Regenerative Biology, Mount Sinai School of Medicine, New York, New York, United States of America; 2 Division of Endocrinology, Metabolism, and Lipid Research, Department of Medicine, Washington University School of Medicine, St. Louis, Missouri, United States of America; 3 Development and Aging Program, NASCR Center, Sanford-Burnham Medical Research Institute, La Jolla, California, United States of America; University of California San Francisco, United States of America

## Abstract

Diets high in carbohydrates have long been linked to progressive heart dysfunction, yet the mechanisms by which chronic high sugar leads to heart failure remain poorly understood. Here we combine diet, genetics, and physiology to establish an adult *Drosophila melanogaster* model of chronic high sugar-induced heart disease. We demonstrate deterioration of heart function accompanied by fibrosis-like collagen accumulation, insulin signaling defects, and fat accumulation. The result was a shorter life span that was more severe in the presence of reduced insulin and P38 signaling. We provide evidence of a role for hexosamine flux, a metabolic pathway accessed by glucose. Increased hexosamine flux led to heart function defects and structural damage; conversely, cardiac-specific reduction of pathway activity prevented sugar-induced heart dysfunction. Our data establish *Drosophila* as a useful system for exploring specific aspects of diet-induced heart dysfunction and emphasize enzymes within the hexosamine biosynthetic pathway as candidate therapeutic targets.

## Author Summary

Heart disease remains the most common source of mortality in the American population. The Western diet has been implicated as an important factor, though the mechanisms by which high levels of dietary sugar and fat contribute to heart failure remain controversial. Here, we use the fruit fly *Drosophila* to explore the role of diet on progressive heart failure. We show that elevated levels of dietary sugar are sufficient to direct progressive heart failure in flies including arrhythmia, reduced fractional shortening, and aspects of fibrosis. We demonstrate the importance of multiple cellular pathways, including the hexosamine biosynthetic pathway, as important for mediating these progressive defects, suggesting a potential avenue towards therapeutics.

## Introduction

Diet-mediated diseases represent an increasing challenge in Western society. Particular attention has focused recently on carbohydrate consumption, which has increased as much as 41% in the past three decades [1]. Dietary sugars have in turn been linked a variety of metabolism-related problems including obesity, insulin resistance, metabolic syndrome, and type 2 diabetes mellitus (T2DM). Heart tissue is thought to be especially sensitive to changes in sugar and insulin flux [2]. Elevated levels of hemoglobin A1c—a measure of long-term blood glucose levels—is an independent risk factor for heart disease in both diabetics and non-diabetics [3]. Regular consumption of sugar-sweetened beverages is associated with a higher risk of coronary heart disease [4]. In addition to coronary heart disease progression of T2DM can lead to diabetic cardiomyopathy, defined as functional or structural defects of myocardial structures in the absence of coronary artery disease or hypertension [5]. As a result, the American Heart Association has recently recommended limiting sources of sugar in the diet [6]. Despite important advances in our understanding of the effects of dietary sugars, our knowledge of the mechanisms that direct sugar-induced heart disease remains incomplete.

*Drosophila melanogaster* provides a useful complement to mammalian models. Their short life span and powerful genetic tools permit detailed *in situ* organ analysis. While flies show important differences from their mammalian counterparts, they also show marked similarities. For example, the fly genome encodes seven insulin-like peptide genes (Dilp 1–7) that activate classical insulin pathway signaling [7], [8]. Ablation of insulin producing cells phenocopied several aspects of type 1 diabetes [9]. Further, recent data have indicated that *Drosophila* is susceptible to diet-mediated metabolic and cardiac organ dysfunctions that are reminiscent of those reported in mammals [10]–[14].

**Figure 1:**
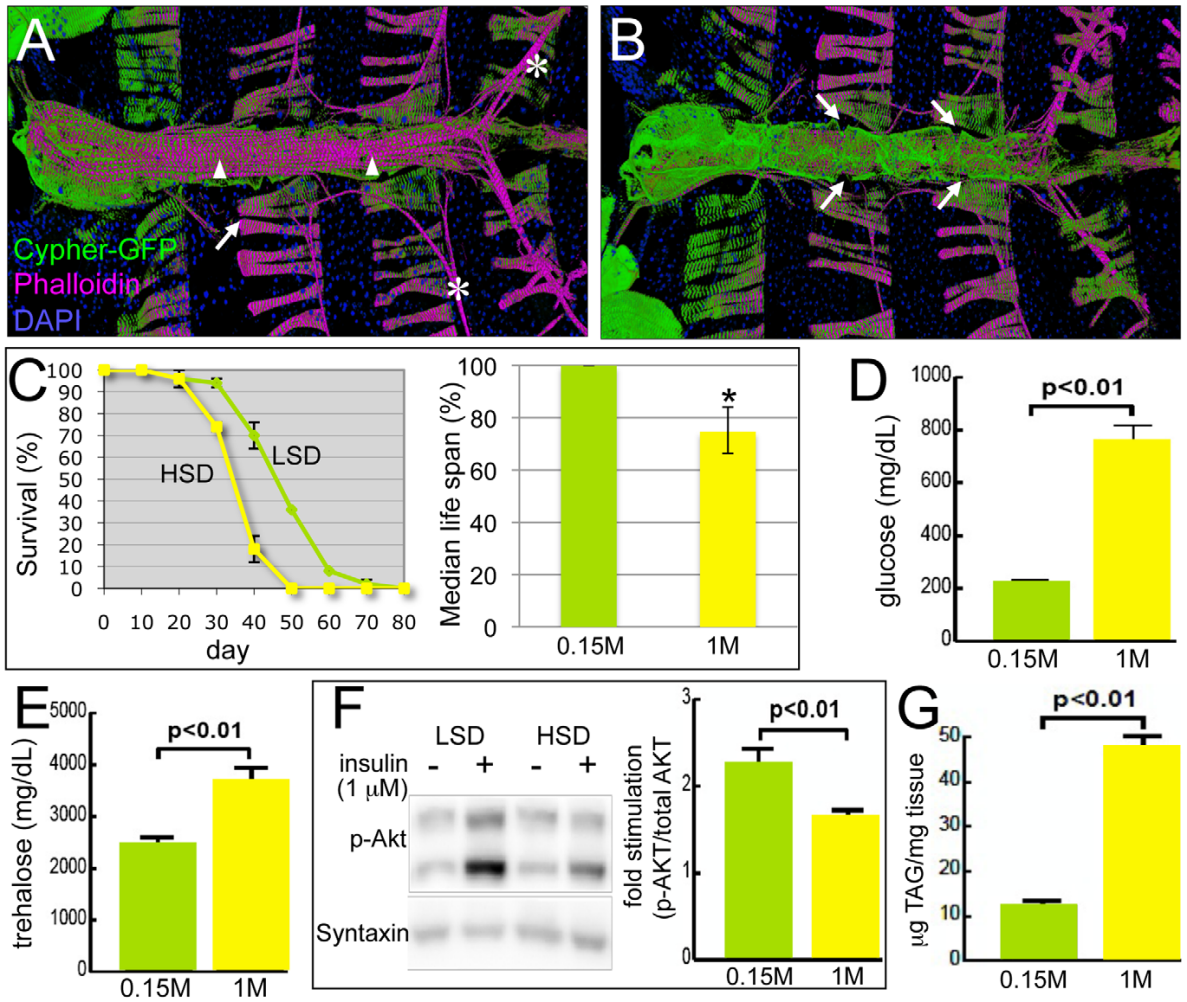
*Drosophila* model of diabetic cardiomyopathy. (A) Ventral view of the heart tube. 3D structures are shown. The heart was stained for F-Actin with phalloidin (magenta) and for nuclei with DAPI; Cypher-GFP (green) labeled Z-lines of myofibers within cardiomyocytes. Arrowheads indicate the non-myocardial longitudinal muscle fibers, asterisks the alary muscles that support the heart, and arrows the abdominal muscles. (B) Dorsal view of the heart tube. Myocardial cells wrap in a circular fashion around the central cavity. Arrows show the ostia through which hemolymph from the abdomen enters into the heart tube and circulates. (C) HSD significantly reduced life span. *w^1118^* male flies were raised in 0.15 M or 1.0 M sucrose diet, food was changed every 2–3 days, and flies were counted every 10 days. HSD-fed flies displayed decreased median life span, 10 days shorter than flies fed an LSD. Mean ± SE are shown; n = 50 total flies in two separate experiments. The estimated median life span of HSD flies was also expressed as the percentage of LSD flies (*p = 7.61E-08 by log rank test). See also Figure S1. (D) Hemolymph glucose concentrations in 3-week-old, control and HSD-fed *w^1118^* adult flies. n≥6. (E) Hemolymph trehalose concentrations in 3-week-old, control and high sucrose-fed *w^1118^* adult flies. n≥6. (F) Bodies from *w^1118^* adults fed LSD or HSD for 3 weeks were treated with insulin (1 µM) or vehicle and visualized using antibodies against *Drosophila* PO_4_-Akt or Syntaxin. n = 10. Bands from Western blot experiments were quantified, and PO_4_-Akt was normalized to Syntaxin as a loading control. (G) Total triglycerides (TAG) were assayed enzymatically in 3-week-old control and high sugar-fed *w^1118^* adult flies, and normalized to weight. n≥12. Mean ± SE are shown. An unpaired, two-tailed t-test was used to derive p-values.

The *Drosophila* heart shows both similarities and differences to the mammalian heart. It is a linear heart tube that is divided into four chambers by rudimentary valve-like structures. *Drosophila* have an open circulatory system with a separate tracheal system used for oxygen transport and their hearts are devoid of coronary arteries [15]–[17]. This separation of oxygen delivery from cardiac pumping function has the advantage that alterations in heart function do not immediately affect viability. Figure 1A demonstrates the ventral view of the heart, showing the longitudinal and alary muscles that cover and stabilize the heart tube, respectively [18]. Figure 1B provides a dorsal view of the heart, showing the myocardial cells that form the heart tube and ostia that provide an entry point for hemolymph. Conserved mechanisms of heart development and function are shared between flies and vertebrates [19]–[22]. Recently, increasingly robust tools have been developed to image the fly heart and to characterize its physiological function [15], [23]–[25]. A *Drosophila* age-related heart disease model has been established and several genes that regulate age-mediated damage have been identified [17], [22], [25]–[28]. Recently, a high fat diet-induced obesity model has been developed in *Drosophila* that leads to severe cardiac malfunction, demonstrating the utility of this genetic system for studying fundamental aspects of organismal metabolic disorders [29]. *Drosophila* is limited as a model for particular aspects of diabetes and diabetic cardiomyopathy including hypertension and vascular defects. Nevertheless, it provides an opportunity to explore specific aspects of metabolic dysfunction and heart function.

Here we develop the *Drosophila* heart as a new model for the study of diet-induced heart dysfunction. Flies were raised on a high-sucrose diet (HSD) to provoke specific aspects of diet-induced metabolic dysfunction including aspects of T2DM. We demonstrate progressive and specific dysfunctions emerging in the hearts of adult flies fed an HSD. We further validate our model by demonstrating that two pathways previously shown to mediate heart dysfunction in mammals— the insulin and P38 MAPK pathways— modulate HSD-induced heart defects in *Drosophila* as well. Finally, we present evidence that dietary sucrose directs heart damage in part by its flux through the hexosamine biosynthetic pathway. Increasing hexosamine flux phenocopied sugar-mediated heart dysfunction and also led to structural damage. Importantly, decreasing pathway activity led to a significant reduction in sucrose-mediated heart damage, suggesting specific enzyme targets that may prove useful for reducing the effects of high dietary sugars on heart function.

## Results

### High dietary sugar shortened *Drosophila* life span and was associated with increased cardiac arrhythmia and heart deterioration

To explore the effects of high carbohydrate feeding and to determine whether *Drosophila* can show diet-mediated heart dysfunction, we compared adults fed an HSD (standard media brought to a final concentration of 1.0 M sucrose) *vs.* control low sucrose diet (LSD, 0.15 M sucrose). One characteristic aspect of metabolic dysfunctions including obesity, T2DM, and heart disease is shortened average life expectancy [30]. Adult flies fed an HSD after eclosion exhibited an average life span reduction of approximately ten days compared to those fed an LSD (Figure 1C and Figure S1; [31]). In addition, they exhibited phenotypes typical of metabolic syndrome and T2DM patients including hyperglycemia, hypertrehalosemia, peripheral resistance to exogenous insulin, and accumulation of triglyceride (Figure 1D–1G). These are similar to HSD effects reported for *Drosophila* larvae [11].

**Figure 2:**
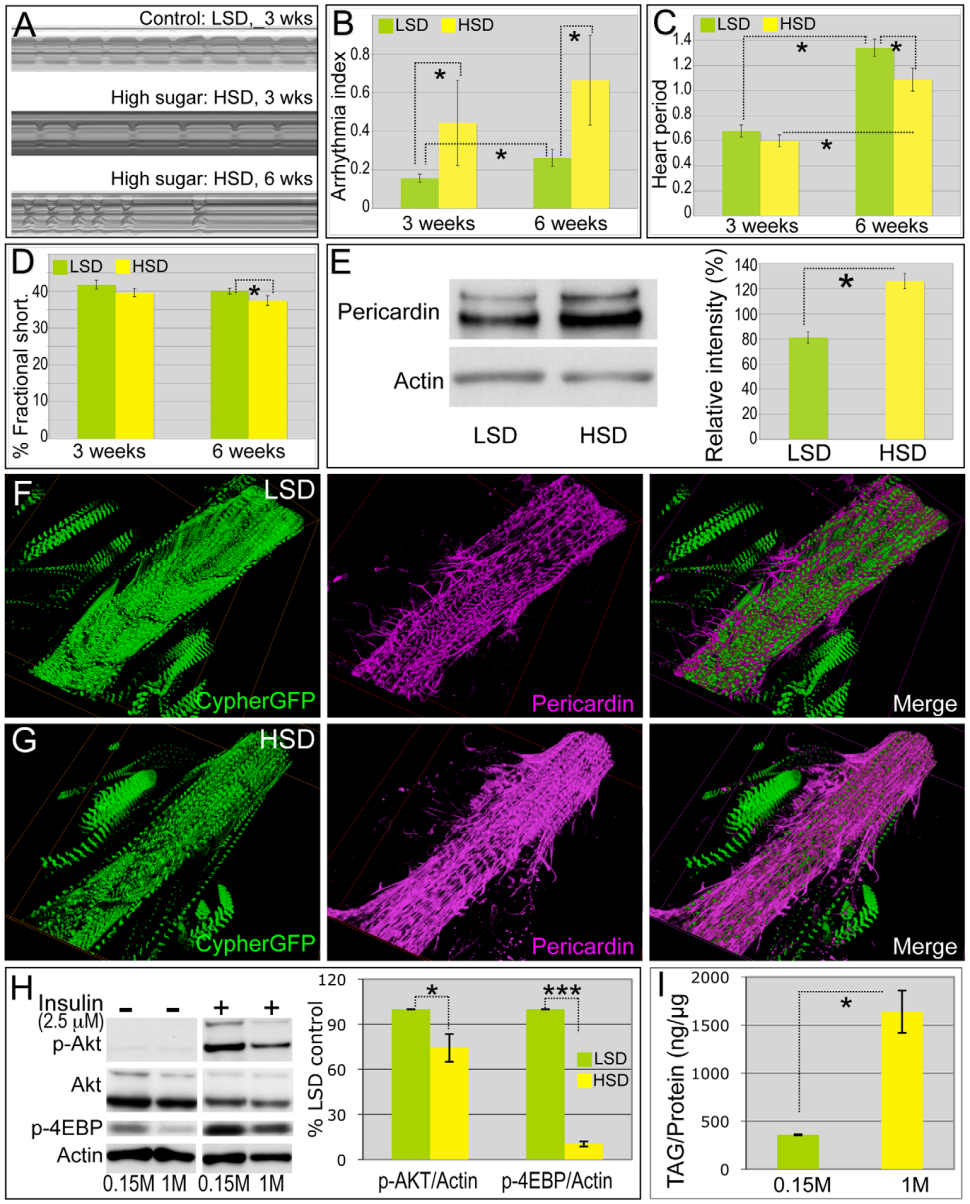
High sucrose shortened *Drosophila* life span and was associated with increased cardiac arrhythmia and heart deterioration. (A) Representative M-mode (5 seconds) from flies fed LSD and HSD. Three-week-old adults fed an HSD showed moderate cardiac arrhythmia; at six weeks arrhythmicity was increased. (B) Arrhythmia index obtained from *w^1118^* flies fed LSD and HSD. Arrhythmias observed in M-mode can be quantified as arrhythmia index, which is the standard deviation of all heart periods in each record normalized to the median heart period for each fly. Mean ± SE are shown. At week three, a significant increase in arrhythmia index was observed in HSD fed flies (0.44) compared to low sucrose fed flies (0.16) (*P = 1.54E-17 by F-test). Arrhythmia index of six-week-old flies increased to 0.66 in HSD and 0.26 in low sucrose diet, respectively (*P = 6.01E-12 by F-test). Data are means ± SE. (C) Heart period of adult files fed low vs. high dietary sucrose. At three weeks of age, no difference was observed between HSD- and LSD-fed flies. Heart period was significantly increased at six weeks of age in both HSD- and LSD-fed flies (*P = 9.64E-06 and 1.44E-09, respectively, by t-test). Interestingly at six weeks of age, heart period of HSD-fed flies was shorter than that of low sucrose fed flies (*P = 0.015 by t-test). Data are means ± SE. (D) Fractional shortening of adult flies fed low *vs.* high dietary sucrose. At three weeks of age, no difference was observed between HSD- and LSD-fed flies; however, fractional shortening was significantly decreased in flies fed HSD- *vs.* LSD-fed (*P = 0.043 by t-test). Data are means ± SE. (E) Quantification of Pericardin level of adult heart by Western blot. Eight hearts from three-week-old LSD- and HSD-fed flies, respectively, were loaded. Pericardin level was detected by a monoclonal antibody against Pericardin, and normalized to Actin level. (F,G) Representative confocal images of three-week-old adult fly hearts expressing Cypher-GFP (posterior A2/anterior A3 segment) and stained with anti-Pericardin (magenta) antibody. Pericardin levels in hearts of flies fed an HSD were increased compared to those fed low sucrose. Note that Pericardin was detected in heart tissue but not abdominal muscles. See also Figure S4. (H) Fly hearts from *w^1118^* adults fed LSD or HSD were treated with insulin (2.5 µM) or vehicle and visualized using antibodies against *Drosophila* PO_4_-Akt, PO_4_-4EBP or Actin, showing the response of the heart to exogenous insulin challenge. n = 3. Bands from Western blot experiments were quantified, and PO_4_-Akt and PO_4_-4EBP were normalized to Actin as a loading control. The ratio of HSD fed flies was then normalized to that of LSD fed flies. PO_4_-Akt and PO_4_-4EBP level were 74.3% and 13.9%, respectively, in HSD-fed flies compared to LSD-fed flies (P = 0.049 and 0.0003, respectively, by t-test). (I) Heart accumulated triglycerides (TAG) were assayed enzymatically in 15 hearts from 3-week-old LSD- and HSD-fed *w^1118^* adult flies, and normalized to protein level. n = 2. (P = 0.028 by t-test). See also Figure S4 and Videos S1, S2, S3.

No significant difference was observed in the survival of high sucrose- and low sucrose-fed flies in the first three weeks (Figure 1C) permitting us to use this period to explore the effects of diet on the heart function of fully viable animals. Figure 2A presents an M-mode for flies raised on LSD. By contrast, flies raised on HSD exhibited a deteriorating heartbeat as manifested by the irregular beating patterns (Figure 2A; Videos S1, S2). Arrhythmias observed by M-mode analysis can be quantified as the arrhythmia index [25]. High dietary sucrose significantly increased the arrhythmia index (Figure 2B; 0.16 LSD *vs.* 0.44 HSD, *P = 1.54E-17 by F-test). However heart period and fractional shortening, a measure of heart contractility, were normal at this age (Figure 2C, 2D).

Sucrose-mediated mortality and heart defects increased significantly as the flies were further aged to six weeks. Less than 20% of adult flies survived past 40 days on an HSD compared to 70% fed an LSD (Figure 1C). Consistent with previous findings [25], flies fed an LSD displayed a moderate increase in their arrhythmia index and heart period as they aged (Figure 2B, 2C). Six-week-old animals exhibited more severe heart phenotypes including fibrillations, asystolic periods and arrhythmia; these defects were significantly enhanced in animals fed an HSD, which also exhibited moderate but consistent defects in fractional shortening (Figure 2A–2D, Video S3). We observed similar effects with a 1.0 M sucrose corn meal-based food (Figure S2), further indicating that increased arrhythmias and decreased fractional shortening are due specifically to dietary sugar.

These defects indicated a potential for emergent structural defects and/or metabolic defects. Cardiac fibrosis is a prominent aspect of heart damage that commonly emerges in diabetic patients [32], [33]. To explore this in flies, we examined collagen levels within the fly heart with an antibody against Pericardin, a *Drosophila* type IV collagen-like protein. Notably, by three weeks of age Pericardin levels and the fibrous extracellular meshwork were significantly and consistently increased within the heart tissue of adult flies fed an HSD, compared to those fed an LSD (Figure 2E–2G).

In addition, the response of the heart to insulin stimulation was defective in HSD-raised flies as manifested by diminished phosphorylation of AKT and 4EBP (Figure 2H and Figure S3). These hearts also accumulated excessive triglyceride (Figure 2I). The observed shortened life span, progressive deterioration of specific heart functions, accumulation of collagen and triglycerides, and aspects of insulin resistance indicate that our HSD feeding regimen represents a useful *Drosophila* model of diet-mediated heart disease. We next explored the molecular mechanisms by which high dietary sucrose led to heart dysfunction.

### P38 and insulin pathways modulate sucrose-induced heart deterioration

**Figure 3:**
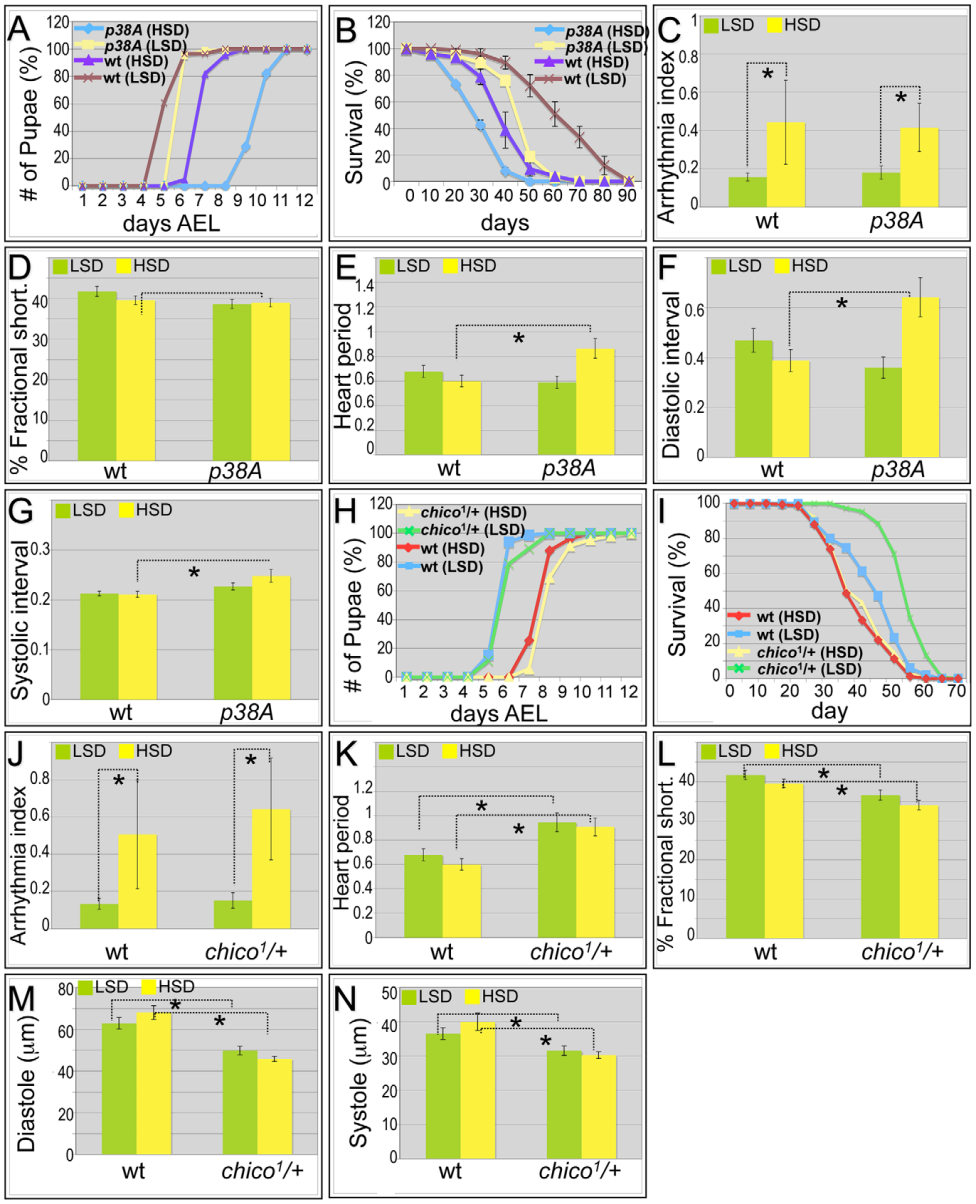
Mutations in *p38A* and *chico* attenuate high sucrose-induced heart deterioration. (A) Mutations in *p38A* enhanced developmental delay of larvae fed an HSD. Eggs were collected onto LSD or HSD food for 16 hours, then permitted to mature at 25°C. Time to pupariation was scored daily. HSD feeding resulted in two days' development delay for *w^1118^* flies. On low sucrose food, *p38A* mutations exhibited a slight delay compared to *w^1118^* controls; an HSD led to a four-day delay to pupariation. (B) Mutant *p38A* flies fed high dietary sucrose exhibited reduced life span. Experiments for both *p38A* mutants and *w^1118^* controls were performed at 22°C due to poor viability of developing *p38A* mutant flies grown at 25°C on an HSD. The average life span of *p38A* flies was ten days shorter than *w^1118^* controls fed an HSD. (C) Loss of *p38A* activity did not alter high dietary sucrose-induced cardiac arrhythmia. Arrhythmia index obtained from *w^1118^* controls and *p38A* mutants. The differences between LSD and HSD both in wild type controls and *p38A* mutants were significant (*P = 1.54E-17, and 1.20E-08, respectively, by F-test). However, there was no difference between *w^1118^* controls and *p38A* mutants. Data are means ± SE. (D) No difference in fractional shortening was observed between *w^1118^* controls and *p38A* mutants grown in either an LSD or an HSD. Data indicate mean ± SE. (E) Heart period was increased in *p38A* mutants fed an HSD. The heart period of *p38A* mutants fed on HSD was 0.86 second, compared to 0.60 second in *w^1118^* controls (*P = 0.008 by t-test). Data indicate mean ± SE. (F) Diastolic interval was increased in *p38A* mutants fed an HSD. Diastolic interval of *p38A* mutants fed an HSD was 0.64 second, compared to 0.39 second in *w^1118^* controls (*P = 0.01 by t-test). Data indicate mean ± SE. (G) Systolic interval was increased in in *p38A* mutants fed an HSD. Systolic interval of *p38A* mutants fed an HSD was 0.25 second, compared to 0.21 second in *w^1118^* controls (*P = 0.02 by t-test). Data indicate mean ± SE. (H) No difference was observed in developmental rates between *w^1118^* controls and *chico^1^*/+ adults fed either an LSD or HSD. Data indicate mean ± SE. (I) Increased life span was observed in *chico^1^*/+ adults compared to *w^1118^* controls when both were fed an LSD. However, the observed average life span in HSD is 35 days in *chico^1^*/+ mutants, the same as *w^1118^* controls. Data indicate mean ± SE. (J) *chico^1^* mutation did not alter high dietary sucrose-induced cardiac arrhythmia. Arrhythmia index obtained from *w^1118^* controls and *chico^1^*/+ mutants. The differences between LSD and HSD both in wild type controls and *chico^1^*/+ mutants were significant (*P = 1.54E-17, and 2.10E-10, respectively, by F-test). However, there was no difference between *w^1118^* controls and *chico^1^*/+ mutants (P = 0.20 by F-test). Data indicate mean ± SE. (K) Heart period was increased in *chico^1^*/+ mutants fed with an LSD or HSD. Heart period of *chico^1^*/+ mutants raised on low sucrose was 0.94 second, compared to 0.68 second of *w^1118^* controls (*P = 0.004 by t-test). Heart period of *chico^1^*/+ mutants raised on an HSD was 0.91 second, compared to 0.60 second of *w^1118^* controls (*P = 0.0008 by t-test). Data indicate mean ± SE. (L) *chico^1^*/+ flies exhibited significantly reduced fractional shortening in both LSD and HSD. Fractional shortening of *chico^1^*/+ mutants raised on low sucrose was 37%, compared to 42% of *w^1118^* controls (*P = 0.006 by t-test). Fractional shortening of *chico^1^*/+ mutants raised on an HSD was 34%, compared to 40% of *w^1118^* controls (*P = 0.001 by t-test). Data are means ± SE. (M) *chico^1^*/+ flies exhibited significantly reduced diastole on both LSD and HSD. Diastole of *chico^1^*/+ mutants raised on low sucrose was 49.9 microns, compared to 62.9 microns of *w^1118^* controls (*P = 0.0009 by t-test). Diastole of *chico^1^*/+ mutants raised on an HSD was 45.8 microns, compared to 68.1 microns of *w^1118^* controls (*P = 2.14E-08 by t-test). Data are means ± SE. (N) Significantly reduced systole of *chico^1^*/+ flies was observed in both LSD and HSD. Systole of *chico^1^*/+ mutants raised on low sucrose was 31.6 microns, compared to 36.6 microns of *w^1118^* controls (*P = 0.03 by t-test). Systole of *chico^1^*/+ mutants raised on an HSD was 30.2 microns, compared to 40.0 microns of *w^1118^* controls (*P = 0.0002 by t-test). Data are means ± SE. See also Videos S4, S5.

P38 is a stress-activated kinase of the mitogen-activated protein kinase (MAPK) superfamily that is activated in models of T2DM. Activation is thought to be related to oxidative stress: for example, the antioxidant lipoic acid increases P38 activation [34] and leads to improved insulin sensitivity in diabetic patients [35], [36]. The *Drosophila* P38 paralog P38A acts as a prototypical stress kinase, and its impairment leads to dysfunction in the presence of various environmental insults [37]. In larvae, metabolic dysfunction can be assayed by assessing the rate of development to pupariation [11]. Larvae homozygous for a null mutation of *p38A* exhibited a slight developmental delay when fed an LSD, requiring an extra day to develop to pupariation; this difference was strongly enhanced when animals were raised on an HSD as larvae, resulting in a four day delay (Figure 3A).

Similarly, the shortened life span exhibited by *p38A* mutant adults was strongly enhanced in the presence of high sucrose, reducing median lifespan by an additional ten days (Figure 3B and Figure S1). The hearts of *p38A(−/−)* flies fed a control LSD exhibited little deviation from wild type (*w^1118^*). However, an HSD led specifically to a strongly increased heart period (decreased heart rate); heart arrhythmia and fractional shortening were unchanged compared to LSD (Figure 3C–3E; Video S4). The HSD-induced increase in heart period was due to significant increases in both the diastolic and systolic intervals (Figure 3F, 3G). The increase in systolic interval length is especially notable in that this parameter is tightly controlled in *w^1118^* flies. These data are consistent with the view that sucrose-mediated deficits in development, longevity, and heart function are normally opposed by P38A activity.

Insulin signaling is a primary regulator of glucose homeostasis. Mis-regulation of pathway components leads to insulin resistance and aspects of metabolic syndrome and diabetes. Reduced insulin signaling can extend life span in worms, flies, and mammals [38]–[40] as well as improve heart function in aging flies [22]. *Drosophila* fed an HSD became progressively insulin-resistant (Figure 1F, Figure 2H), raising the question of whether systemic reduction of insulin signaling would protect heart function damaged by HSD.

To address these questions we tested the effects of reducing the functional copy number of the insulin receptor substrate (IRS) ortholog *chico* in the presence of high sucrose feeding. Utilizing a null *chico* allele, genotypically *chico^1^/*+ heterozygous adults showed the same developmental delay in LSD *versus* HSD as did *w^1118^* (Figure 3H). As anticipated, *chico^1^/*+ adults lived 13 days longer on an LSD (Figure 3I and Figure S1). By contrast, removing one functional copy of *chico* did not extend life span on an HSD: half of the animals died at approximately day 35, similar to *w^1118^* controls fed an HSD (Figure 3I and Figure S1). Metabolic assays indicated that, compared to *w^1118^* controls, *chico^1^/*+ heterozygous adults had similar triglyceride levels but higher hemolymph glucose levels (Figure S5).

When examining the heart function of *chico^1^/*+ heterozygotes fed an LSD, we observed a slight increase in heartbeat arrhythmicity compared to wild type controls although the difference did not reach statistical significance (Figure 3J; Video S5). By contrast, the heart period of *chico^1^/*+ heterozygotes displayed significant differences on both diets when compared to controls: 0.94 seconds when fed LSD and 0.91 seconds in HSD compared to 0.67 and 0.60 seconds, respectively, observed in *w^1118^* flies (Figure 3K; Video S5). Other heart parameters were affected as well. Fractional shortening of *chico^1^/*+ hearts was significantly decreased both in high sugar food and low sugar food (Figure 3L), indicating that the pumping ability of the *chico^1^/*+ heart was compromised. The heart size of *chico^1^/*+ mutant flies was dramatically decreased as demonstrated by decreased diastolic and systolic diameter (Figure 3M, 3N). We validated previous studies that found no reduction in body size of *chico^1^/*+ mutants ([41]; not shown), indicating that the decrease in heart size is likely due to a heart-specific effect reduced *chico* activity. We conclude that high sucrose feeding of *chico^1^/*+ heterozygotes leads to compromised heart performance by strongly reducing heart period and fractional shortening, which may contribute to an overall reduction in life span. Thus, the beneficial effects of reduced insulin signaling (*e.g.*, to extend lifespan) strongly depend on the dietary composition [14] and are lost when the animal consumes high dietary sugar.

### Activation of the hexosamine pathway led to heart dysfunction

**Figure 4:**
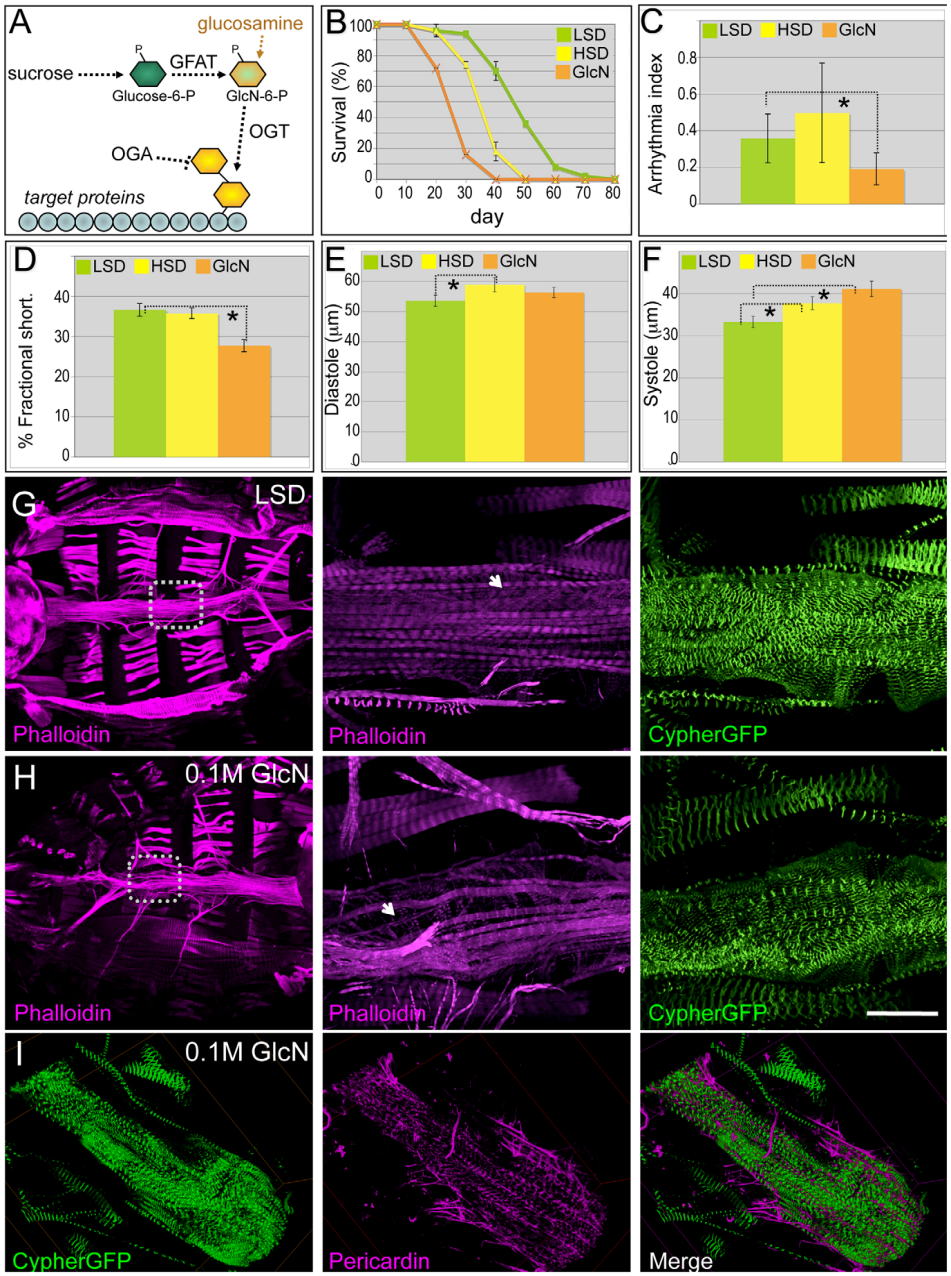
Dietary glucosamine shortened life span and induced cardiac dysfunction. (A) Hexosamine biosynthesis pathway (HBP). The two rate limiting enzymes GFAT and OGT convert glucose to a O-GlcNAc residue that is then targeted to protein substrates; this residue is removed by β-N-acetylglucosaminidase (OGA). Glucosamine bypasses GFAT and increases HBP flux. (B) Dietary glucosamine significantly reduced life span: the average life span of flies fed 0.1 M glucosamine was 25 days compared to 35 days on an HSD and 48 days on a (control) LSD. Data are means ± SE (n = 2 experiments, 25 flies per experiment). (C) Glucosamine-fed flies displayed decreased arrhythmia. Arrhythmia index was obtained from wild type (*Canton S*) in LSD, HSD, and glucosamine diet. Glucosamine diet significantly reduced arrhythmia index (*P = 0.006 by F-test). Note *Canton S* flies fed with low sucrose showed slightly higher arrhythmia than *w^1118^* flies fed with low sucrose. Data are means ± SE. (D) Glucosamine reduced fractional shortening in three-week-old adult flies (*P = 0.001 by t-test). Data are means ± SE. (E) Unlike high dietary sucrose (*P = 0.042 by t-test), dietary glucosamine did not change the diastolic diameter (P = 0.32 by t-test). Data are means ± SE. (F) Diets supplemented with glucosamine (*P = 0.0009 by t-test) or HSD (*P = 0.04 by t-test) increased systolic diameter, indicating that changes in fractional shortening are due to changes in systolic diameter. Data are means ± SE. (G,H) Dietary glucosamine led to heart structure defects. F-Actin in the heart was visualized with phalloidin (red) and Cypher-GFP (green) to label the Z-lines of myofibers within cardiomyocytes (magnification = 10×). Insets magnify the boxed regions to show the myofibers at higher magnification (63×). Size bar = 250 µm. (I) Representative confocal images of a three-week old Cypher-GFP fly heart from an animal fed 0.1 M glucosamine. Visualization with anti-Pericardin antibody (magenta) indicated decreased Pericardin levels. Compare with the control shown in Figure 2E. See also Video S6.

Several metabolic pathways are thought to mediate glucose's ability to provoke metabolic dysfunction, insulin resistance, and T2DM including the polyol, AGE, PKC, and hexosamine biosynthetic pathways [42]. The outcome of excess flux through the hexosamine biosynthetic pathway remains unclear: high levels are thought to damage pancreatic beta cells through O-linked glycosylation [43], but the physiologic significance of hexosamine flux *vs.* oxidative stress has been questioned [44]. Glucose flux through the hexosamine pathway utilizes the key enzymes glutamine-fructose-6-phosphate transaminase (GFAT) and O-linked beta-N-acetylglucosamine transferase (OGT), yielding the sugar modification UDP-GlcNAc that regulates a broad range of target proteins (Figure 4A). Increased hexosamine flux and O-GlcNAc levels have been implicated in the impaired relaxation of isolated cardiomyocytes, blunted response to angiotensin II and phenylephrine, hyperglycemia-induced cardiomyocyte apoptosis, and endothelial and vascular cell dysfunction [45]. Targeting OGT to the mouse liver led to significant insulin resistance and related metabolic defects through its interaction with phosphoinositides [46].

To specifically access hexosamine pathway activity we fed flies the hexose sugar glucosamine (Figure 4A). Knockdown of GFAT in a subset of tissues (*dot>gfat-IR*) led to early larval lethality; this lethality was rescued by low levels (0.02–0.04 M) of glucosamine, validating its targeting of hexosamine flux (Figure S6). Diets supplemented with moderate levels of glucosamine (0.1 M) significantly shortened life span: the median life span for glucosamine-fed flies was 25 days compared to animals fed HSD (35 days) or LSD (48 days; Figure 4B). Hearts of flies fed 0.1 M glucosamine did not exhibit elevated arrhythmia; rather, heart rhythmicity was more highly ordered than flies fed an HSD or even, surprisingly, an LSD (Figure 4C; Video S6). However, fractional shortening was significantly decreased to 28%, compared to 37% in flies grown in control food (Figure 4D). The decrease in fractional shortening is likely due to systolic dysfunction: the systolic diameter increased significantly while the diastolic diameter did not change relative to LSD controls (Figure 4E, 4F). Meanwhile, the heart response to exogenous insulin was diminished in glucosamine-fed flies compared to controls (Figure S7).

The impaired contractile ability of the heart observed in glucosamine-fed flies was reminiscent of the heart dilation phenotype reported in mice with cardiomyocyte-restricted knockout of the insulin receptor [47]. In these mice heart dilation was coupled with structural defects, prompting us to examine the heart structures of flies fed control, high sucrose, and glucosamine-supplemented diets. Phalloidin was used to visualize integrity of the cytoskeleton and Cypher-GFP was used to visualize the sarcomeric Z-line patterns within the myofibrils of the contractile myocardium. Adult flies fed an LSD for three weeks retained correctly aligned and organized myofibril organization (Figure 4G). However, three-week old adults fed 0.1 M glucosamine exhibited disorganized longitudinal myofibrillar structures as well as disorganized transverse myofibrils of the underlying myocardium (Figure 4H). The fibers themselves often displayed signs of separation and gaps between them and the myofibrils were thinner than controls. We observed similar but milder defects in three-week adult flies fed 1.0 M sucrose; phenotype penetrance was also lower (data not shown). This data suggests that at least some of the functional defects we observed in hearts of adult flies fed glucosamine can be traced to emergent structural defects.

We did note a consistent difference between high sugar and glucosamine-supplemented diets. Unlike HSD-fed flies, which exhibited increased Pericardin accumulation (Figure 2G), flies fed a glucosamine-supplemented diet consistently exhibited decreased Pericardin levels within the heart (Figure 4I), compared to LSD-fed flies (Figure 2F).

### Knockdown of hexosamine pathway components led to improved heart function in adults fed a high sucrose diet

The role of the hexosamine biosynthetic pathway on heart homeostasis is poorly understood in the context of the whole animal. In mice, knockout of OGT resulted in embryonic lethality indicating that hexosamine flux is required for normal development [48]. However, reducing hexosamine pathway-mediated O-GlcNAc protein modification did not prevent insulin resistance in 3T3-L1 adipocytes [49]. Whole animal data in *C. elegans* has not fully illuminated these issues: reduced *ogt* activity lowered dauer formation induced by a temperature sensitive insulin-like receptor (*daf-2*) mutant, while reduced activity of the negative regulator *oga* augmented dauer formation. However, both *ogt* and *oga* mutants showed carbohydrate and lipid metabolism alterations that were similar to changes associated with mammalian insulin resistance [50], [51].

**Figure 5:**
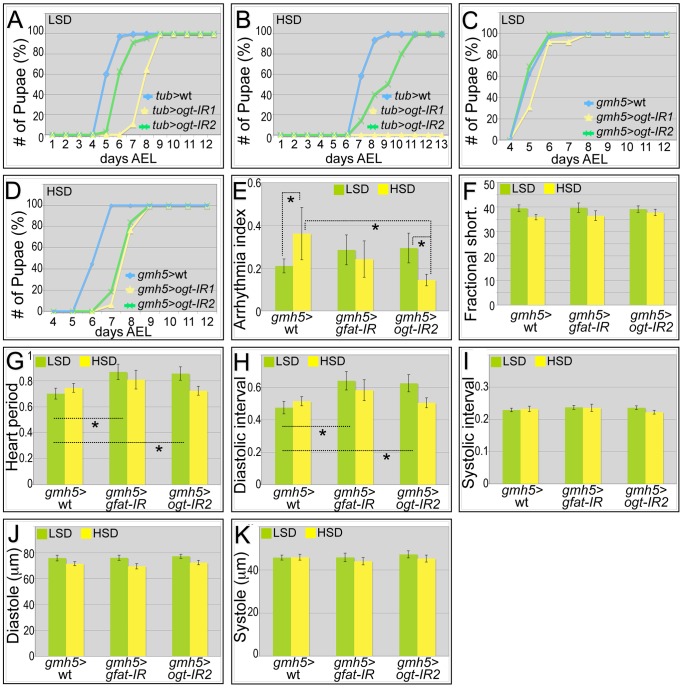
Genetic manipulation of the hexosamine biosynthetic pathway. (A) Global knockdown of OGT with two separate *ogt-IR* lines by *tub-gal4* led to a delay in development to pupariation in larvae fed an LSD. (B) This delay was further enhanced in the presence of an HSD: in particular, knockdown of OGT by *tub>ogt-IR1* led to lethality. (C) Heart-specific knockdown of OGT (*gmh5-gal4*) led to little or no effect on development rate in the presence of control food. (D) By contrast, heart-specific knockdown of OGT (*gmh5>ogt-IR*) led to a 1.5 day developmental delay in the presence of high dietary sucrose. (E) Heart-specific knockdown of OGT or GFAT decreased high sugar induced arrhythmia. Arrhythmia index was somewhat increased—though not statistically significantly— in flies with a heart-specific knockdown of OGT or GFAT flies raised in low sucrose diet compared to wild type controls. However, when raised on an HSD, heart-specific knockdown of OGT or GFAT led to decreased arrhythmia compared to wild type controls; especially for OGT knockdown flies, the decrease in arrhythmia index was significant (*P = 5.57E-08 by F-test). (F) Heart specific knockdown of GFAT or OGT did not alter fractional shortening. Fractional shortening of wild type controls, *gmh5>gfat-IR* flies and *gmh5>ogt-IR2* flies are shown. Data represent means ± SE. (G) Heart period did not change in heart specific knockdown of GFAT or OGT flies raised on an HSD. However, when raised in low sucrose diet, they displayed increased heart period (*P = 0.02 for both GFAT or OGT knockdown by t-test). (H) Diastolic interval did not change in heart specific knockdown of GFAT or OGT flies raised on an HSD. However, when raised in low sucrose diet, they showed increase of diastolic interval (*P = 0.03 for GFAT knockdown and 0.04 for OGT knockdown by t-test). (I) Systolic interval did not change in heart specific knockdown of GFAT or OGT flies raised on an LSD or HSD. (J) Diastole did not change in heart specific knockdown of GFAT or OGT flies raised on an LSD or HSD. (K) Systole did not change in heart specific knockdown of GFAT or OGT flies raised on an LSD or HSD. See also Videos S7, S8, S9.

Our data indicate that increased activation of the hexosamine biosynthetic pathway leads to an adverse impact on adult *Drosophila* cardiac function. We therefore examined whether dietary sucrose does indeed act at least in part through hexosamine flux. Removing one genomic copy of OGT (*sxc/+*) led to increased hyperglycemia and significant heart dysfunction including increased arrhythmia and decreased fractional shortening in animals fed an LSD or HSD diet (Figure S8). Global knockdown of *Drosophila gfat* utilizing inverted-repeat (IR) mediated RNA-interference proved lethal early in development (data not shown). Knockdown of *ogt* with two separate *ogt-IR* lines—using a strong ubiquitous driver (*tub>ogt-IR*) or weaker cardiac-specific driver (*gmh5>ogt-IR*)—permitted viability though we observed a developmental delay that was enhanced in the presence of high dietary sucrose (Figure 5A–5D).

Heart specific knockdown of OGT (*gmh5>ogt-IR*) or GFAT (*gmh5>gfat-IR*) displayed increased arrhythmicity in flies fed a low sucrose diet (Figure 5E), though Pericardin levels were unaffected (Figure S9). Importantly, this arrhythmicity was decreased in *gmh5>gfat-IR* and more strongly decreased in *gmh5>ogt-IR* flies fed an HSD (Figure 5E, and Videos S7, S8, S9). This result indicates that the effects of dietary sucrose on arrhythmia are mediated at least in part through GFAT and OGT. Although the heart period and diastolic interval of *gmh5>ogt-IR* and *gmh5>gfat-IR* flies were increased in control food, they were not significantly different from wild type controls fed an HSD: fractional shortening, systolic interval, diastole and systole remained unchanged (Figure 5F–5K). Of note, we failed to observe a significant diet-induced size change in *gmh5-GAL4* control hearts, suggesting GAL4 itself may have a subtle effect on heart size (Figure 5J–5K).

Together, our data suggest that the hexosamine biosynthetic pathway plays a significant role in the development of multiple aspects of diet-mediated heart dysfunction in *Drosophila*: increased pathway activity led to heart damage while decreased function was protective for flies raised on high dietary sucrose.

## Discussion

### Heart function in a *Drosophila* T2DM model

Several mechanisms have been proposed to account for the pathogenesis of diet-induced heart dysfunction. A primary injury may reflect alterations in energy substrate supply and utilization. For example, a mammalian diabetic cardiomyocyte model exhibited defects in glycolysis and glucose oxidation [52] and shifts to β-oxidation of free fatty acids that led to metabolism-based dysfunction of the heart [53]–[58]. Further structural changes arose from myocyte cell death, accumulation of collagen and fibrosis [59].

In our study we observed a subset of these phenotypes. By three weeks, adult flies fed an HSD exhibited detectable heart arrhythmia, a phenotype that increased with age. The contractility of the heart was not detectably impaired by three weeks, as fractional shortening was not decreased. However the size of the heart—as assessed by both diastole and systole— was increased (Figure 4E, 4F), reminiscent of the dilated cardiomyopathy reported for diabetic patients [60]. In addition, contractility decreased with age, phenocopying more advanced aspects of heart disease including diabetic cardiomyopathy. Importantly we also observed progressive fibrosis-like collagen accumulation within the heart, a central aspect of progressive heart disease in mammals (Figure 2E–2G).

### Chico and P38 effects on heart function were diet-dependent

Previous work in mammals has demonstrated that insulin signaling is important for heart function. It regulates cardiac size, metabolism, and contractile protein expression [61], and cardiomyocyte-specific insulin receptor knockout mice exhibited impaired heart function [62]. Signaling through the IRS adaptor protein regulates mitochondrial gene expression, mitochondrial function and cardiomyocyte survival [63]. In this work, we demonstrate a similar requirement for the insulin pathway: in the presence of high dietary sugar, reduced activity of the IRS ortholog *chico* led to a decrease in fractional shortening that in turn contributed to progressive heart failure. This may help explain why reduced insulin pathway activity—which acts to extend lifespan on a normal diet—leads to shorter lifespan in the presence of high dietary sugar: reduced insulin signaling diminishes the ability of the animal to accommodate non-optimal diets.

P38 interacts with the insulin signaling pathway in specific contexts. Phosphorylation of P38 was responsive to insulin stimulation in the retina but not the liver in a mouse model of diabetes [64]. In rat aortic vascular smooth muscle cells, high glucose and chronic insulin treatment that mimicked hyperglycemia and hyperinsulinemia impaired iNOS induction by acute insulin treatment and was associated with sustained P38 activation. Blocking P38 pathway with the chemical inhibitor SB-203580 restored iNOS induction [65]. In contrast, in rat hearts insulin has been shown to protect myocardial contractility [66]; this protection requires P38 MAPK phosphorylation of Hsp27 [67]. In our experimental paradigm P38 proved to be required for full protection from high dietary sugar. This may reflect its role in protecting from cellular stress: *Drosophila* lacking P38A function are vulnerable to specific environmental stresses including heat shock, oxidative stress and starvation [37].

### Hexosamine biosynthetic pathway is a candidate therapeutic target

The hexosamine biosynthetic pathway is critical for cell function and loss of the pathway effector enzyme OGT in mice led to embryonic lethality [68]. We found that reducing activity of either OGT or GFAT specifically in the adult *Drosophila* heart led to improved heart function as demonstrated by reduced incidence of arrhythmias in the presence of an HSD.

Our data are mostly consistent with results in other model systems. Elevated glucose, glucosamine, or over-expressed OGT led to increased O-linked glycosylation of nuclear proteins and loss of OGT-impaired cardiomyocyte calcium cycling. In contrast, over-expressing the pathway inhibitor OGA reversed sugar-induced heart defects including calcium transients [69]. O-linked glycosylation of mitochondrial proteins impaired mitochondrial function in cardiac myocytes exposed to high glucose, an effect that was reversed by over-expression of OGA [70]. Our data are not consistent with recent observations from rat cardiac fibroblasts, which demonstrated that flux through the hexosamine biosynthetic pathway induced collagen expression [71]: in our whole animal paradigm, glucosamine decreased Pericardin levels (Figure 4I).

Deleting OGT function specifically within the hearts of infarcted mice significantly exacerbated cardiac dysfunction [72]. We also observed moderately impaired heart function in cardiac knockdown of OGT and GFAT of flies fed an LSD. In contrast, cardiac knockdown of OGT and GFAT of flies fed an HSD was strongly protective of heart dysfunction compared to control flies on an HSD (Figure 5E–5H). Reducing GFAT or OGT activity did not detectably alter Pericardin levels (Figure S8) suggesting that GFAT or OGT acts on other components to improve the heart function of HSD-fed flies. The heart dysfunction observed in both reduced and increased hexosamine flux suggests that a correct balance is required to maintain normal heart function. Based on the observation that reducing hexosamine biosynthetic pathway activity rescued several aspects of heart dysfunction in our *Drosophila* model, our work supports GFAT, OGT and OGA as candidate therapeutic targets.

## Materials and Methods

### Fly stocks

*cn1 P{ry11}chico1/CyO*; *ry506*, P38A and *P{w[+mC] = Dot-GAL4.K}43A*, y[1] w[*] ([73], cat. #6903) flies were obtained from the Bloomington *Drosophila* Stock Center. *w^1118^* control and transposable P-element insertions *w^1118^*; *P{UAS-OGT-IR,w[+]}* and *w^1118^*; *P{UAS-GFAT-IR,w[+]}* transgenic flies were obtained from the Vienna *Drosophila* RNAi Center [74]. *gmh5-GAL4* was obtained from Sanford-Burnham Institute [22].

### Fly food recipes

All flies, from embryo stage, were raised on Bloomington standard cornmeal food [75]. After eclosion, adults were transferred to LSD or HSD food, which were made based on Bloomington semi-defined medium [76], with adjustment of sugar concentration. 100 ml food contains agar (1 g), yeast (8 g), yeast extract (2 g), peptone (2 g), MgSO_4_ (200 µl of 1 M solution), CaCl_2_ (340 µl of 1 M solution), propionic acid (600 µl), mold inhibitor (1000 µl), and sucrose (5.13 g = 0.15 M for LSD, 34.2 g = 1.0 M for HSD). 0.1 M glucosamine was supplemented to the LSD food to make the glucosamine food.

### Hemolymph glucose and trehalose measurements

Hemolymph was pooled from 35–45 adult flies to obtain 1 µl for assay. Hemolymph was diluted 1∶10 before assay. Glucose was measured by adding to 99 µl of Thermo Infinity Glucose Reagent (TR15321) in a 96 well plate, then incubated for 3 minutes at 37°C and read immediately at 340 nm. Trehalose was measured using the same reagent after 24 hours of incubation, at which point endogenous trehalase has completely digested the trehalose to produce free glucose. Glucose standards were treated simultaneously and used to quantify the sugar levels in hemolymph.

### Western blotting

For insulin sensitivity assays, 3-week-old LSD- or HSD-fed adults were dissected open to expose the body cavity to treatments. Recombinant human insulin (Sigma I0259) or dilution buffer (10 mM HEPES) were added to Schneider's medium and incubated at room temperature for 15 minutes. Heads were removed and bodies were homogenized, sample buffer added, and tissue used to generate Western blots. For heart insulin sensitivity assays, same challenging procedure was applied, and followed by harvesting the heart tissue and directly lysed for Western blotting. Cell Signaling antibodies against *Drosophila* PO_4_-Akt (#4054) were used to detect Akt phosphorylation, anti-pan-Akt (#4691) for pan-Akt, anti-PO_4_-4EBP (#2855) for 4EBP phosphorylation, and anti-Pericardin (DSHB EC11) for Pericardin. Anti-actin (DSHB JLA20) or anti-syntaxin (DSHB 8C-3) was used as a loading control. Secondary antibodies were from Santa Cruz.

### Lipid measurements

For whole body TAG, 6 animals were homogenized in PBS+0.1% Tween and heated for 5 minutes at 65°C to inactivate lipases. 2 µl of this homogenate was mixed with 198 µl of Thermo Infinity Triglyceride reagent and analyzed per the manufacturer's instructions. Triglyceride standards were treated simultaneously and used to quantify TAG levels. The heart TAG was measured according to [29].

### Optical heartbeat analysis of adult *Drosophila* hearts

Heart parameters were analyzed from high speed optical recordings of exposed hearts from adult *Drosophila* (Ocorr et al, PNAS 2007; Ocorr and Vogler, JoVE 2009). High speed digital movies of beating hearts were taken using a Hamamatsu EMCCD 9300 camera (Hamamatsu, Inc.; 100–140 frames per second) and Simple PCI image capture software (Hamamatsu, Inc.).

Heart period, heart diameters, percent fractional shortening and M-modes were determined from the movies using a previously described computer algorithm [24]. Heart periods were defined as the time between the ends of two consecutive diastolic intervals. Regarding M-modes, a single line of pixels bisecting the ventricle was identified in one frame of the movie and the same line of pixels was electronically excised from each subsequent frame and aligned horizontally to generate a snapshot of the movement of the heart edges (y axis) over time (x axis). Diastolic and systolic diameters were obtained as output from the MatLab-based program (Mathworks, Natick, MA). Arrhythmias observed in M-mode can be quantified as an arrhythmia index, which is the standard deviation of all heart periods in each record normalized to the median heart period for each fly.

### Fluorescent staining and imaging

Adult hearts were dissected in artificial *Drosophila* hemolymph (ADH) and rhythmic beating was confirmed in oxygenated ADH. Hearts were fixed with 4% formaldehyde in PBS for 30 minutes on ice, and washed 2×15 minutes with PBSTx at room temperature. Abdominal cuticle and fat body were removed and specimens were incubated with 10 µl primary antibody (Anti-Pericardin 1∶1000, Developmental Studies Hybridoma Bank) diluted in 1× PAXDG (1× PBS with 1% BSA, 0.3% Triton-X, 0.3% sodium deoxycholate, and 5% normal goat serum) overnight at 4°C, washed 3×15 minutes with 10 µl PBSTx at room temperature, incubated in secondary antibody in 1× PAXDG for 2 hrs (in certain cases with Alexa594-phalloidin (1∶200, Molecular Probes)), washed, and mounted. Confocal images were obtained with a Leica DM5500. Experimental and controls were imaged using identical microscope settings. 3D structures were reconstituted from confocal stacks using Amira software.

### Developmental delay

Eggs were collected onto low or high sucrose food for 16 hours, then permitted to mature at 25°C. Time to pupariation was scored daily.

### Survival

Male flies were raised in control and experimental diet that was changed every 2–3 days, and flies were counted every 5 or 10 days. n = 50 total flies in two separate experiments. Experiments for both p38A mutants and *w^1118^* controls were performed at 22°C due to poor viability of developing p38A mutant flies grown at 25°C on an HSD. Median lifespans were estimated using linear interpolation over scoring intervals. Confidence intervals for estimated medians were computed numerically using the bias-corrected and accelerated bootstrap method with 100000 bootstrap replicates. Pairwise equality of survival distributions was tested by a log-rank test, specifically by an asymptotic permutation test on Sun's log-rank scores for the interval-censored data. Computations were carried out in R (version 2.15.1), a language and environment for statistical computing, using the interval and boot packages.

### Glucosamine rescues lethality due to GFAT1 knockdown

To prepare Dot-Gal4/+; UAS-GFAT^RNAi^/+ eggs, unmated females homozygous for Dot-Gal4 were crossed with males homozygous for UAS- GFAT^RNAi^. Dot-Gal4 expresses in a range of tissues including hemocytes, pericardial cells, the gut, and the salivary glands.

## Supporting Information

Figure S1 Median Lifespan under different dietary and genetic combinations. (A) Life span table of all genotypes under different dietary and log rank statistics. (B) Median Lifespan under different dietary and genetic combinations. Data represent the estimated median lifespan for the indicated genotypes under low sucrose (LSD), high sucrose (HSD), or low sucrose supplemented with glucosamine (LSD+GlcN), see Materials and Methods for details. Error bars show confidence intervals. All groups are significantly different (p<0.05) from their controls and from each other with the exception of Chico −/+ on high sucrose which is not significantly different from w^1118^ flies on either low or high sucrose. *indicates the experiments were done at 22°C, otherwise 25°C.
(TIF)

Figure S2 Heart parameters of flies on corn meal food. (A) Arrhythmia index obtained from WT flies fed corn meal in the absence or presence of 1.0 M sucrose. (B) Fractional shortening obtained from WT flies fed corn meal in the absence or presence of 1.0 M sucrose.
(TIF)

Figure S3 Western blot of PO_4_-4EBP response to insulin stimulation. (A) Western blot of the heart response to insulin stimulation using PO_4_-4EBP antibody (3 experiment repeats). (B) Normalized PO_4_-4EBP level to its loading control Actin. (C) Normalized PO_4_-4EBP of HSD fly compared to normalized p-4EBP on LSD fly.
(TIF)

Figure S4 Confocal images of three-week old *w^1118^* fly hearts. Hearts stained with anti-Pericardin (magenta) and anti-phalloidin (green) antibodies. Associated with Figure 2.
(TIF)

Figure S5 Metabolic characterization of *chico^1^*/+ adults. Associated with Figure 3. (A) Hemolymph glucose concentrations in 3-week-old, control and high sucrose-fed *w^1118^* and *chico^1^*/+ adult flies. n≥3. (B) Total triglycerides (TAG) were assayed enzymatically in 3-week-old control and high sugar-fed *w^1118^* and *chico^1^*/+ adult flies, and normalized to protein level. n≥3.
(TIF)

Figure S6 (A) Glucosamine rescues lethality due to GFAT1 knockdown. Dot-Gal4/+; UAS-GFAT^RNAi^/+ eggs were collected before hatching and transferred to low-sucrose food supplemented with 0, 0.02 M, 0.03 M, or 0.04 M glucosamine, and incubated at 25°C. Survival to pupariation increased linearly with glucosamine dosage over this range. This functionally validates both the on-target effect of the RNAi construct and also the ability of dietary glucosamine to boost intracellular glucosamine directly, or at least independent of GFAT function. Associated with Figure 4. (B) Verification of knockdown of hexosamine pathway. *Drosophila* nephrocytes of wild type or OGT knockdown flies were stained with WGA targeting O-glycosylation. Associated with Figure 4.
(TIF)

Figure S7 Insulin response of the hearts from *w^1118^* adults fed control, high sugar or glucosamine diets for 3 weeks. Bands from Western blot experiments were quantified, and PO_4_-Akt was normalized to actin as a loading control; n = 3. PO_4_-Akt level was 74.3% or 57.9%, respectively, in high sugar or glucosamine diets fed flies compared to LSDs. Associated with Figure 4.
(TIF)

Figure S8 Characterization of *sxc/+* adults. Associated with Figure 5. (A) Removing one functional copy of Drosophila *ogt* led to hyperglycemia. Hemolymph glucose concentrations in 3 week-old, control and high sucrose-fed *w^1118^* and *sxc^6^/+* adult flies. n≥3. (B) Removing one copy of OGT led to increased heart arrhythmia. Arrhythmia index obtained from *w^1118^* controls, *sxc^1^*/+ and *sxc^6^*/+ mutants raised on HSD (*P = 0.036 and 2.55E-11, respectively, by F-test). Note that the arrhythmia index for *w^1118^* is higher than typical in this experiment. (C) Removing one copy of OGT led to decreased fractional shortening. Fractional shortening obtained from *w^1118^* controls, *sxc^1^*/+ and *sxc^6^*/+ mutants raised on HSD. (*P = 0.014, and 0.008, respectively, by t-test).
(TIF)

Figure S9 Reduced *ogt* or *gfat* did not alter Pericardin levels. Average of three experiments. Associated with Figure 5.
(TIF)

Video S1 Live imaging of the heart of a wild type adult fly that was fed a low-sucrose diet for three weeks after eclosion. Associated with Figure 2.
(AVI)

Video S2 Live imaging of the heart of a wild type adult fly that was fed a high-sucrose diet for three weeks after eclosion. Associated with Figure 2.
(AVI)

Video S3 Live imaging of the heart of a wild type adult fly that was fed a high-sucrose diet for six weeks after eclosion. Associated with Figure 2.
(AVI)

Video S4 Live imaging of the heart of a *p38A* mutant adult fly that was fed a high-sucrose diet for three weeks after eclosion. Associated with Figure 3.
(AVI)

Video S5 Live imaging of the heart of a *chico^1^* adult fly that was fed a high-sucrose diet for three weeks after eclosion. Associated with Figure 3.
(AVI)

Video S6 Live imaging of the heart of a wild type adult fly that was fed a 0.1 M-glucosamine-supplemented low-sucrose diet for three weeks after eclosion. Associated with Figure 4.
(AVI)

Video S7 Live imaging of the heart of a *GMH5* heterozygous adult fly that was fed a low-sucrose diet for three weeks after eclosion. Associated with Figure 5.
(AVI)

Video S8 Live imaging of the heart of a *GMH5>GFAT-IR* adult fly that was fed a high-sucrose diet for three weeks after eclosion. Associated with Figure 5.
(AVI)

Video S9 Live imaging of the heart of a *GMH5>GFAT-IR* adult fly that was fed a low-sucrose diet for three weeks after eclosion. Associated with Figure 5.
(AVI)
