# Supplementary figures and images for: A Drosophila Model of High Sugar Diet-Induced Cardiomyopathy
Source: PLoS Genet. 2013 Jan 10;9(1):e1003175. doi: 10.1371/journal.pgen.1003175 (PMC3542070; doi:10.1371/journal.pgen.1003175)

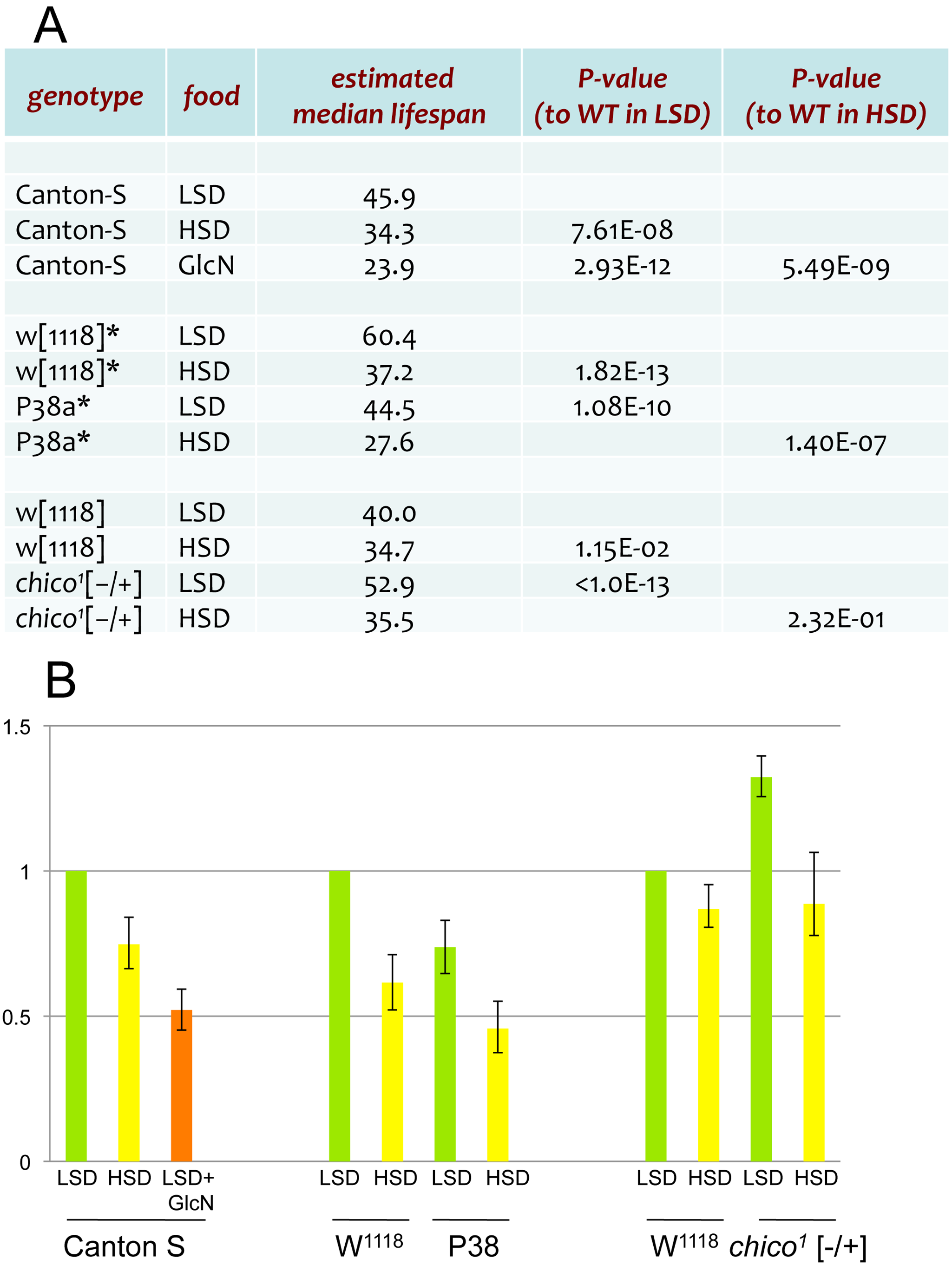

Supplement: Figure S1 — Median Lifespan under different dietary and genetic combinations. (A) Life span table of all genotypes under different dietary and log rank statistics. (B) Median Lifespan under different dietary and genetic combinations. Data represent the estimated median lifespan for the indicated genotypes under low sucrose (LSD), high sucrose (HSD), or low sucrose supplemented with glucosamine (LSD+GlcN), see Materials and Methods for details. Error bars show confidence intervals. All groups are significantly different (p<0.05) from their controls and from each other with the exception of Chico −/+ on high sucrose which is not significantly different from w1118 flies on either low or high sucrose. *indicates the experiments were done at 22°C, otherwise 25°C. (TIF) [file pgen.1003175.s001.tif]

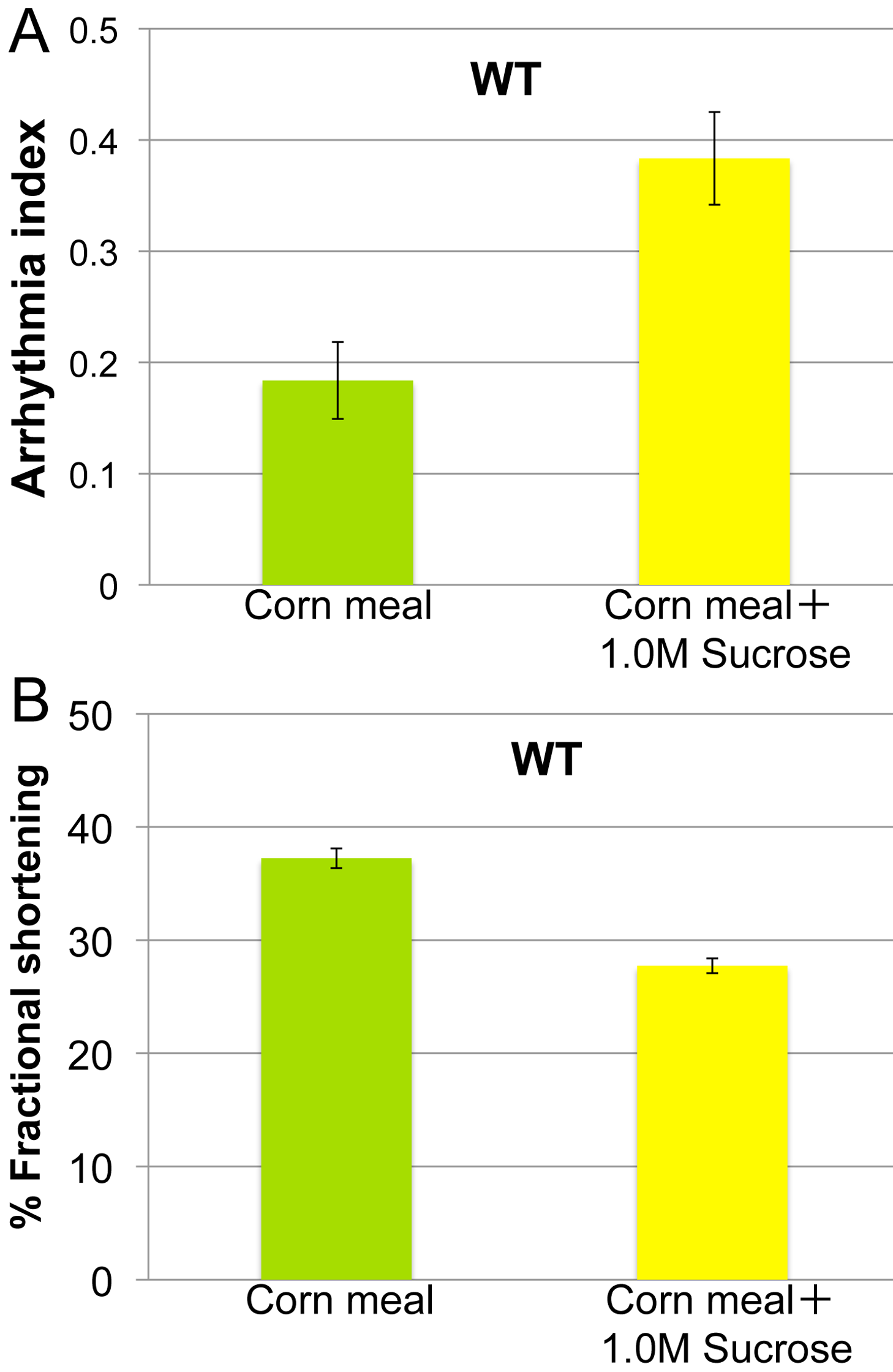

Supplement: Figure S2 — Heart parameters of flies on corn meal food. (A) Arrhythmia index obtained from WT flies fed corn meal in the absence or presence of 1.0 M sucrose. (B) Fractional shortening obtained from WT flies fed corn meal in the absence or presence of 1.0 M sucrose. (TIF) [file pgen.1003175.s002.tif]

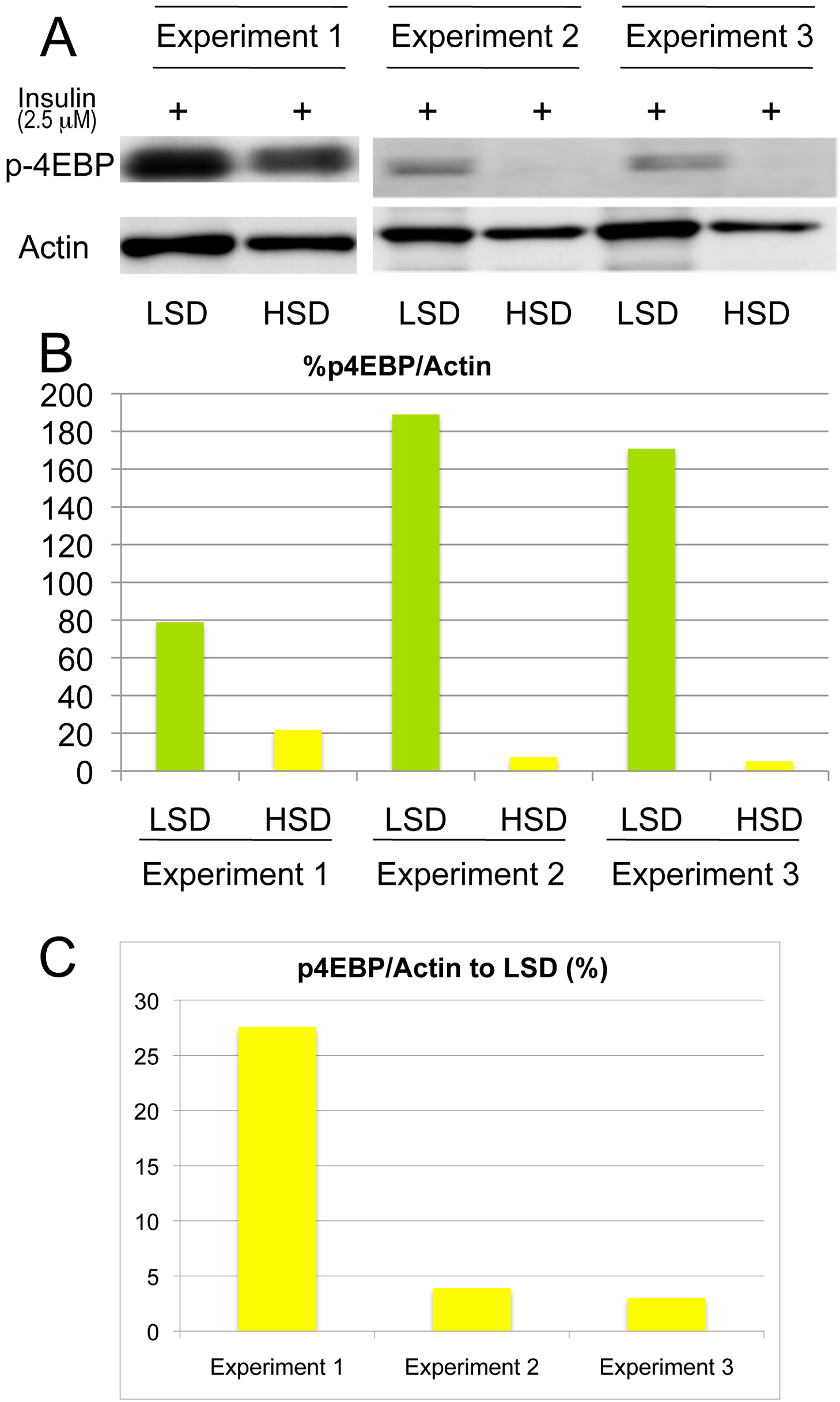

Supplement: Figure S3 — Western blot of PO4-4EBP response to insulin stimulation. (A) Western blot of the heart response to insulin stimulation using PO4-4EBP antibody (3 experiment repeats). (B) Normalized PO4-4EBP level to its loading control Actin. (C) Normalized PO4-4EBP of HSD fly compared to normalized p-4EBP on LSD fly. (TIF) [file pgen.1003175.s003.tif]

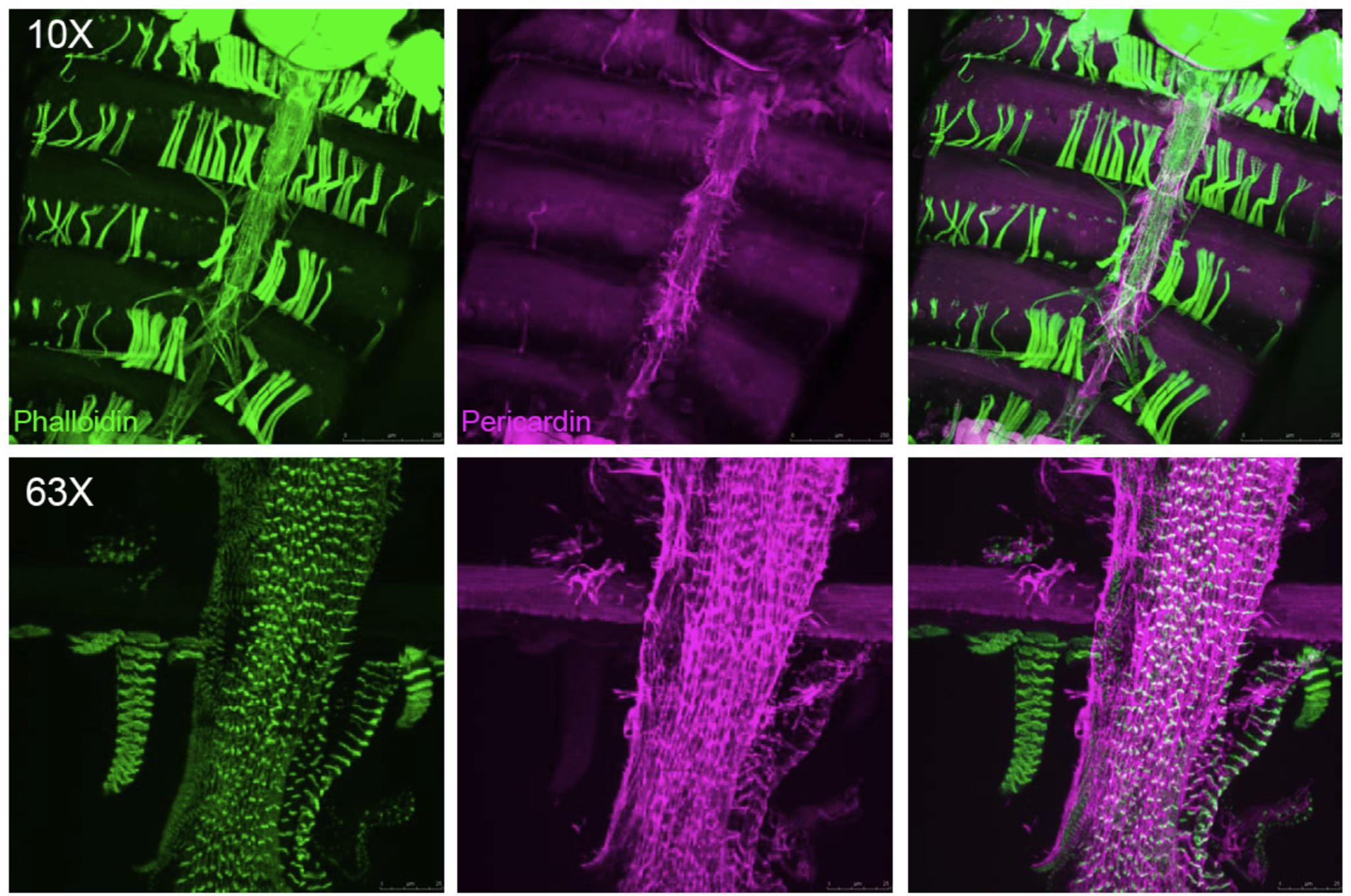

Supplement: Figure S4 — Confocal images of three-week old w1118 fly hearts. Hearts stained with anti-Pericardin (magenta) and anti-phalloidin (green) antibodies. Associated with Figure 2. (TIF) [file pgen.1003175.s004.tif]

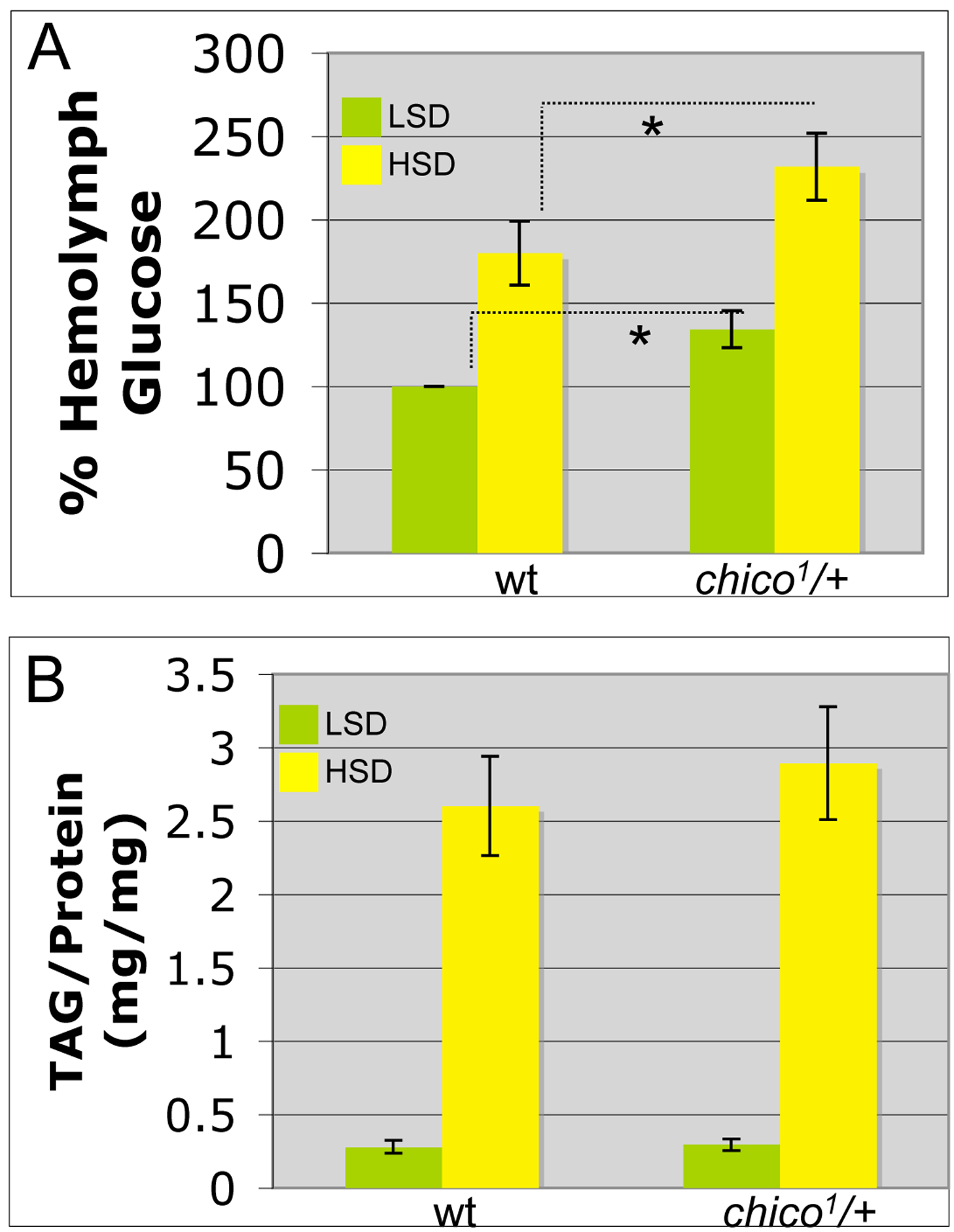

Supplement: Figure S5 — Metabolic characterization of chico1/+ adults. Associated with Figure 3. (A) Hemolymph glucose concentrations in 3-week-old, control and high sucrose-fed w1118 and chico1/+ adult flies. n≥3. (B) Total triglycerides (TAG) were assayed enzymatically in 3-week-old control and high sugar-fed w1118 and chico1/+ adult flies, and normalized to protein level. n≥3. (TIF) [file pgen.1003175.s005.tif]

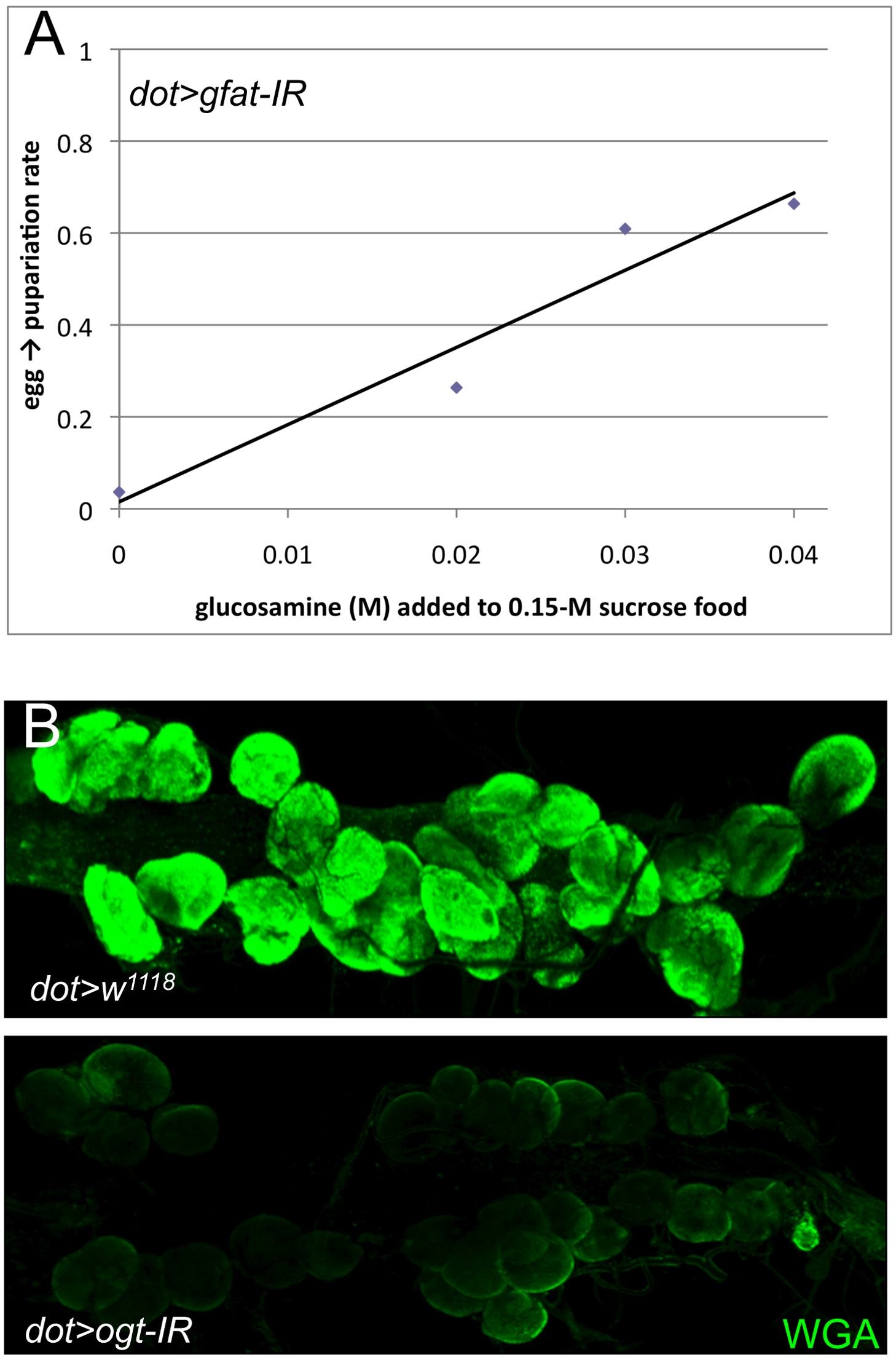

Supplement: Figure S6 — (A) Glucosamine rescues lethality due to GFAT1 knockdown. Dot-Gal4/+; UAS-GFATRNAi/+ eggs were collected before hatching and transferred to low-sucrose food supplemented with 0, 0.02 M, 0.03 M, or 0.04 M glucosamine, and incubated at 25°C. Survival to pupariation increased linearly with glucosamine dosage over this range. This functionally validates both the on-target effect of the RNAi construct and also the ability of dietary glucosamine to boost intracellular glucosamine directly, or at least independent of GFAT function. Associated with Figure 4. (B) Verification of knockdown of hexosamine pathway. Drosophila nephrocytes of wild type or OGT knockdown flies were stained with WGA targeting O-glycosylation. Associated with Figure 4. (TIF) [file pgen.1003175.s006.tif]

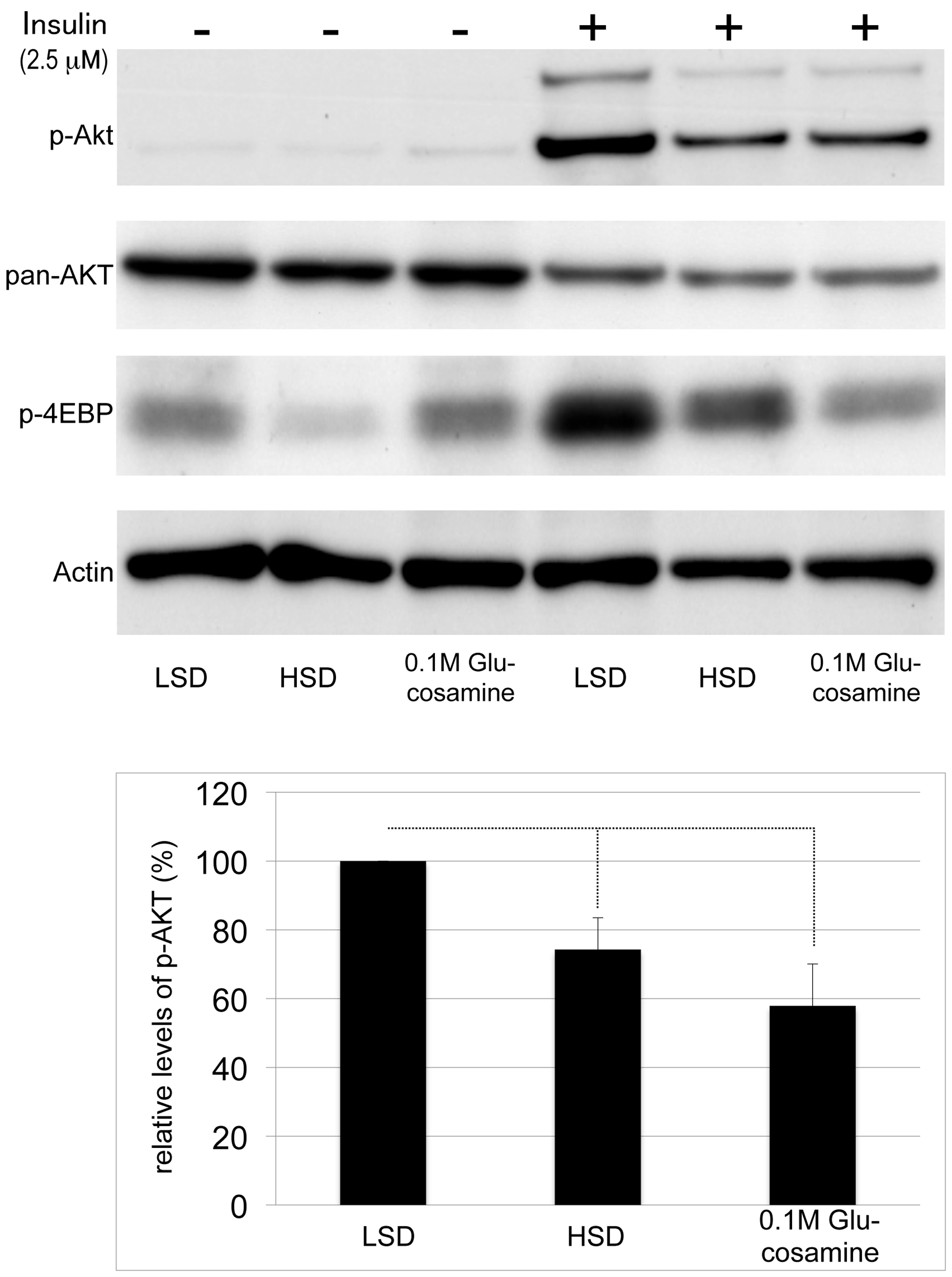

Supplement: Figure S7 — Insulin response of the hearts from w1118 adults fed control, high sugar or glucosamine diets for 3 weeks. Bands from Western blot experiments were quantified, and PO4-Akt was normalized to actin as a loading control; n = 3. PO4-Akt level was 74.3% or 57.9%, respectively, in high sugar or glucosamine diets fed flies compared to LSDs. Associated with Figure 4. (TIF) [file pgen.1003175.s007.tif]

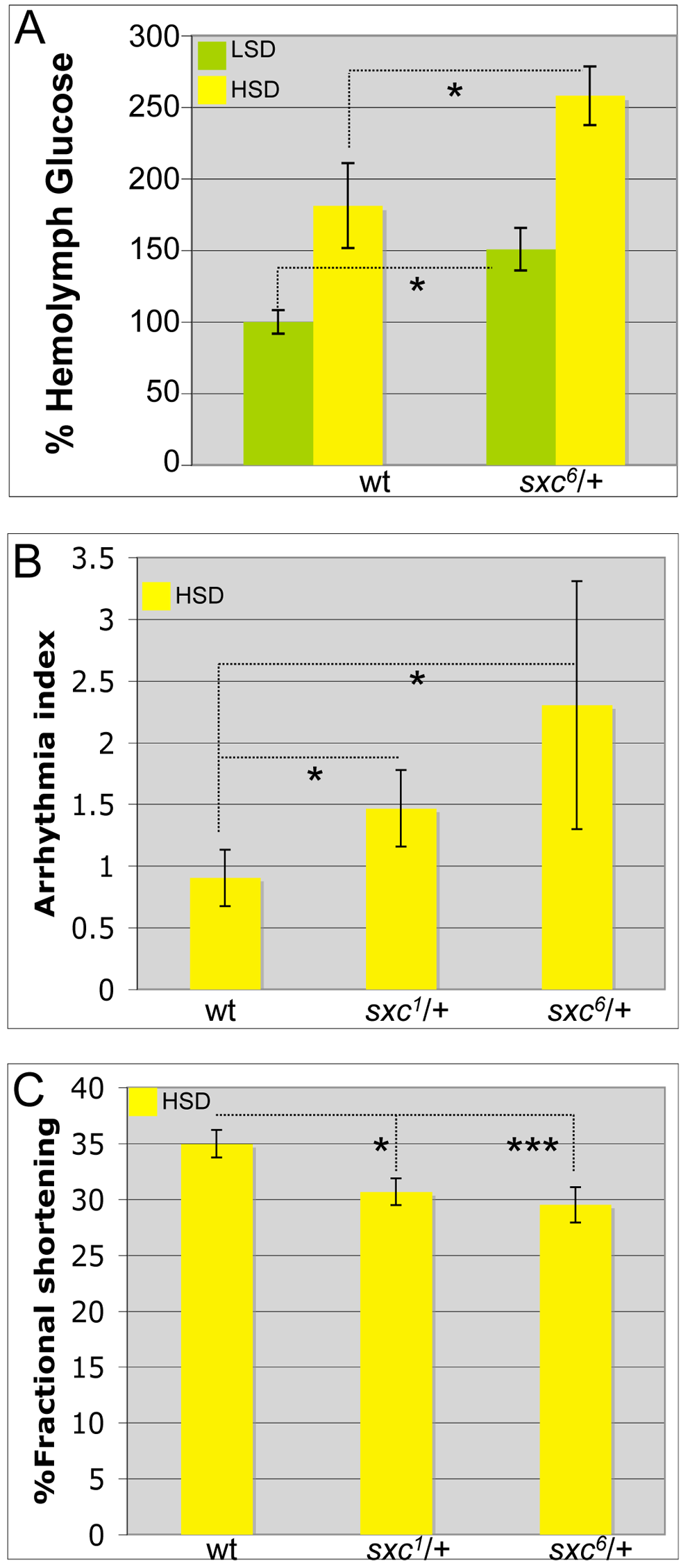

Supplement: Figure S8 — Characterization of sxc/+ adults. Associated with Figure 5. (A) Removing one functional copy of Drosophila ogt led to hyperglycemia. Hemolymph glucose concentrations in 3 week-old, control and high sucrose-fed w1118 and sxc6/+ adult flies. n≥3. (B) Removing one copy of OGT led to increased heart arrhythmia. Arrhythmia index obtained from w1118 controls, sxc1/+ and sxc6/+ mutants raised on HSD (*P = 0.036 and 2.55E-11, respectively, by F-test). Note that the arrhythmia index for w1118 is higher than typical in this experiment. (C) Removing one copy of OGT led to decreased fractional shortening. Fractional shortening obtained from w1118 controls, sxc1/+ and sxc6/+ mutants raised on HSD. (*P = 0.014, and 0.008, respectively, by t-test). (TIF) [file pgen.1003175.s008.tif]

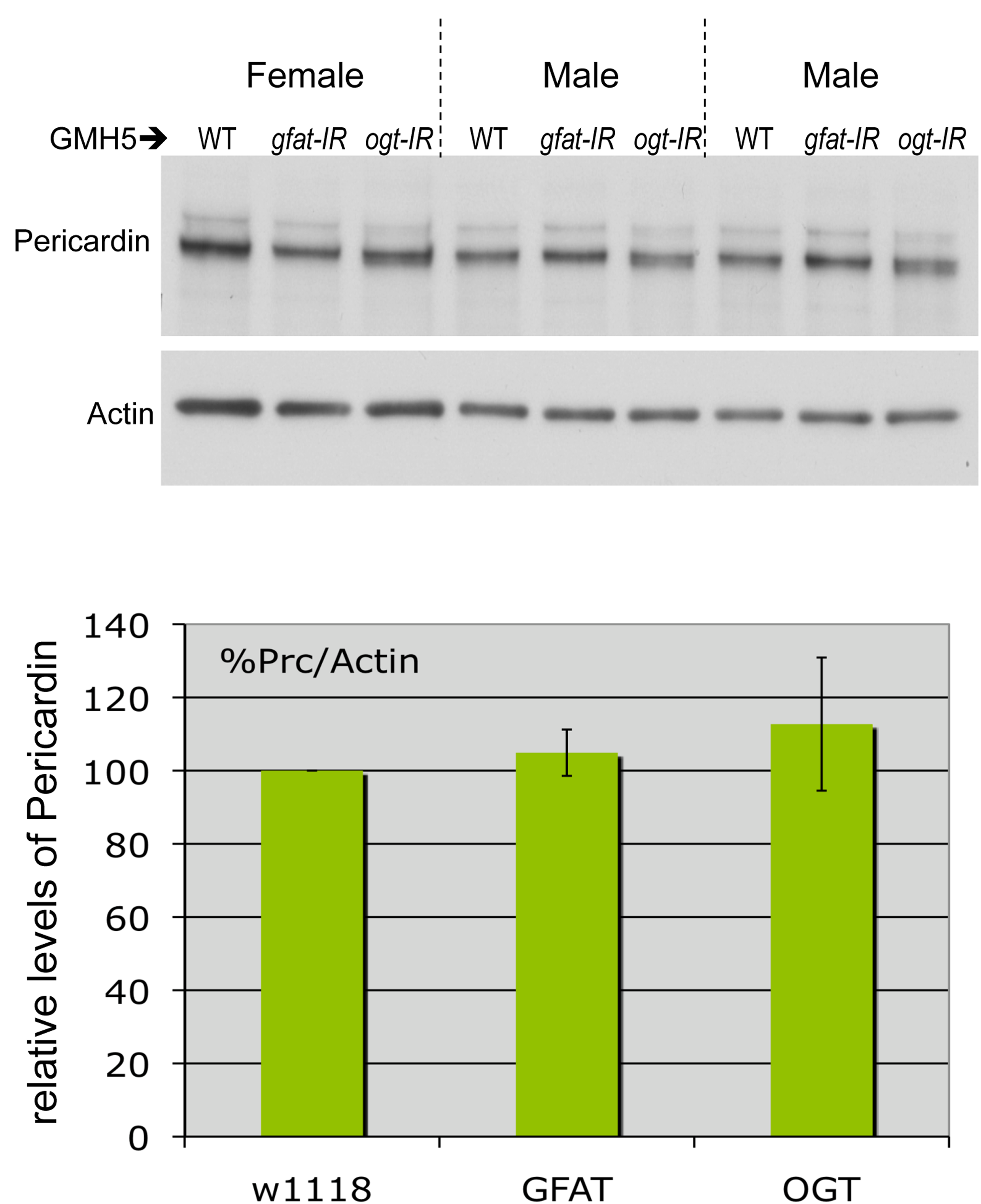

Supplement: Figure S9 — Reduced ogt or gfat did not alter Pericardin levels. Average of three experiments. Associated with Figure 5. (TIF) [file pgen.1003175.s009.tif]
